# The Regulation of Corticofugal Fiber Targeting by Retinal Inputs

**DOI:** 10.1093/cercor/bhv315

**Published:** 2016-01-06

**Authors:** Eleanor Grant, Anna Hoerder-Suabedissen, Zoltán Molnár

**Affiliations:** Department of Physiology, Anatomy and Genetics, University of Oxford, Oxford OX1 3QX, UK

**Keywords:** cortical layer V, VIa, VIb, corticothalamic projections, cross-hierarchical plasticity, dorsal lateral geniculate nucleus, monocular enucleation

## Abstract

Corticothalamic projection systems arise from 2 main cortical layers. Layer V neurons project exclusively to higher-order thalamic nuclei, while layer VIa fibers project to both first-order and higher-order thalamic nuclei. During early postnatal development, layer VIa and VIb fibers accumulate at the borders of the dorsal lateral geniculate nucleus (dLGN) before they innervate it. After neonatal monocular enucleation or silencing of the early retinal activity, there is premature entry of layer VIa and VIb fibers into the dLGN contralateral to the manipulation. Layer V fibers do not innervate the superficial gray layer of the superior colliculus during the first postnatal week, but also demonstrate premature entry to the contralateral superficial gray layer following neonatal enucleation. Normally, layer V driver projections to the thalamus only innervate higher-order nuclei. Our results demonstrate that removal of retinal input from the dLGN induces cortical layer V projections to aberrantly enter, arborize, and synapse within the first-order dLGN. These results suggest that there is cross-hierarchical corticothalamic plasticity after monocular enucleation. Cross-hierarchical rewiring has been previously demonstrated in the thalamocortical system (Pouchelon et al. 2014), and now we provide evidence for cross-hierarchical corticothalamic rewiring after loss of the peripheral sensory input.

## Introduction

The thalamocortical system comprises multiple sets of connections between the thalamus and cortex providing substrates for peripheral sensory relays and cortico-cortical communication (Sherman and Guillery 1998). Cortical projections to the thalamus originate from 3 cortical layers. Layer V provides driver input to thalamic relay cells in higher-order thalamic nuclei from collateral branches of fibers that project to subcortical targets such as the superior colliculus (SC) in the midbrain (Deschênes et al. 1994). The layer V corticothalamic projection mediates a trans-thalamic cortico-cortical relay (Sherman and Guillery 1996, 1998; Jones 2001, 2002). Layer VI is made up of 2 sublayers, layer VIa and VIb, both of which project to the thalamus, but it is unclear what their relative contribution is to the overall projection pattern. Layer VI neurons project to all thalamic nuclei, providing modulatory feedback input to thalamic relay cells in both first-order nuclei and higher-order thalamic nuclei (Sherman and Guillery 1996, 1998). First-order thalamic nuclei relay peripheral sensory information to primary cortical areas; higher-order nuclei relay cortico-cortical information. This cortico-cortical feedback includes direct excitatory input and indirect inhibitory input, via the thalamic reticular nucleus (TRN) and interneurons in the dorsal lateral geniculate nucleus (dLGN; Olsen et al. 2012; Lam and Sherman 2013).

The murine corticothalamic projections start to grow toward the thalamus during embryogenesis, but the functional connections are not established until the first week of postnatal development, a period in which intrinsic genetic mechanisms and peripheral neuronal activity interact to regulate circuit formation (Sur and Leamey 2001; Sur and Rubenstein 2005; Yamamoto and López-Bendito 2012). The timing and route taken by developing corticothalamic projections has been described (Jacobs et al. 2007; Grant et al. 2012; Brooks et al. 2013; Seabrook et al. 2013). Distinguishing developmental patterns specific to each cortical population, however, has been difficult due to the lack of layer-specific markers for these axons. In this study, we use 3 transgenic mouse lines to label the 3 subpopulations selectively (Gensat 2013). The Rbp4-Cre line expresses Cre-recombinase in layer V cortical neurons (Gong et al. 2003, 2007). The Ntsr1-Cre line expresses Cre-recombinase in layer VI cortical neurons (Olsen et al. 2012). Both neurons and neurites become labeled when these lines are crossed with the stop-floxed tdTomato line R26R-Ai14 (Madisen et al. 2010). The Golli-τ-eGFP mouse expresses eGFP mostly in layer VIa and VIb cells and neurites, but there is some small additional labeling within layer V as well (Jacobs et al. 2007; Piñon et al. 2009). Our study shall examine the timing and specificity of these corticofugal projections from different cortical layers into the thalamus.

Activity driven by peripheral sensory organs is important for the development of numerous features of the central nervous system including topography, eye-specific segregation, and periphery-related cytoarchitectural/connectivity patterns (Penn et al. 1998; Hooks and Chen 2006; Piñon et al. 2009; Rebsam et al. 2009, 2012).

Retinal fibers invade the dLGN and the superficial gray layer of the SC before corticofugal fibers do (Drager and Hubel 1975; Shatz and Rakic 1981; Godement et al. 1984; Jacobs et al. 2007; Grant et al. 2012; Seabrook et al. 2013). Retinal fibers transmit spontaneous waves of activity, which propagate across the retina, to both the dLGN and superficial gray layer for over a week before eye opening in mice (Mooney et al. 1996; Ackman et al. 2012).

We have investigated whether retinal input regulates the ingrowth of cortical fibers to the shared targets in the dLGN and SC. We performed monocular enucleation on new-born Golli-τ-eGFP and Ntsr1-Cre::tdTomato mice to study the role of the retinogeniculate input on ingrowth of layer VIa and VIb cortical axons to dLGN. To distinguish between the effects of the loss of the retinal ganglion cell axons and the loss of retinal neuronal activity, we disrupted the spontaneous retinal activity patterns with intraocular injections of potent cholinergic agonist epibatidine on the Golli-τ-eGFP mouse (Penn et al. 1998; Sun et al. 2008; Rebsam et al. 2009, 2012; Ackman et al. 2012). Separately, we performed monocular enucleation on new-born Rbp4-Cre::tdTomato mice to assess whether retinal fibers regulate the timing of layer V corticofugal fiber ingrowth into the superficial gray layer of the SC. Following the removal of retinal driver inputs to the dLGN, we additionally demonstrate that layer V fibers in the Rbp4-Cre::tdTomato mouse aberrantly invade the enucleated dLGN, suggesting that there can be cross-hierarchical rewiring of cortical input to the thalamus.

Briefly, we summarize our results as follows: Subcortically projecting neurons in the different cortical layers project into the thalamus in patterns determined by their position in the first-order, higher-order thalamic nuclei hierarchy. This pattern is established at the time of projection outgrowth and first innervation of the thalamus. Patterned retinal input regulates the timing of corticofugal fiber ingrowth to the dLGN and the SC. Loss of retinal input accelerates layer VI ingrowth to the dLGN and layer V ingrowth to the SC, and causes layer V to innervate the dLGN aberrantly.

## Materials and Methods

### Experimental Animals

All experiments were approved by the local ethical review committee of the University of Oxford and conducted in accordance with personal and project licenses in accordance with the Animals (Scientific Procedures) Act, 1986 (ASPA).

Golli-τ-eGFP, Rbp4-Cre, Ntsr1-Cre, and Ai14-R26R-tdTomato mice were maintained on a standard diet and light cycle in accordance with ASPA and were mated in the local animal facility, University of Oxford, UK. Rbp4-Cre and Ntsr1-Cre mice were mated with Ai14-R26R-tdTomato to label Cre-expressing cells.

### Postnatal Fixed Tissue Collection

P0–P10 mice were anesthetized with an intraperitoneal injection of pentobarbitone (150 mg/kg, Pentoject, Animal Care Ltd, UK) and transcardially perfused with 0.1 M phosphate-buffered saline (PBS). Mice that received epibatidine intraocular injection were killed by cervical dislocation. Brains were dissected and fixed in 4% paraformaldehyde (TAAB, Reading, UK) for 24 h at 4 °C. Brains were stored at 4 °C in 0.1 M PBS with 0.05% sodium azide (Sigma, Gillingham, UK; PBSA). Brains were embedded in 4% agarose and sectioned coronally to 50 µm on a vibrating microtome (Leica, VT1000S) before analysis. Sections were counterstained with 5 µg/mL 4′,6-diamidino-2-phenylindole (DAPI) in PBS for 5 min.

### Monocular Enucleation

P0 mouse pups were anesthetized by hypothermia for 2–3 min until unresponsive to pressure on the rear paw. A 5-mm stab microsurgical knife was used to make a small incision in the skin over the left eye, which was opened slightly using forceps. Sharp forceps were used to separate the eye from surrounding tissue and the optic nerve was cut. The entire left eye, including lens, retina, and retinal pigmented epithelium, was removed. After surgery, the animals were warmed until pink and moving normally before all pups were returned to the home cage.

### Intraocular Injections of Epibatidine or Sterile Saline

Pups were anesthetized using hypothermia as above and kept ice cold for the duration of surgery. A small incision over the anterior half of the left eye was made using a 5-mm stab microsurgical knife and using a platinum wire a small hole was made in the sclera in the anterior quarter of the eye, avoiding the lens. Using a stereotaxic frame, a 10-µL Hamilton Syringe (Hamilton Company USA, Nevada, USA) filled with epibatidine was guided through the hole into the vitreous humor. The epibatidine was injected steadily over 60 s to prevent mechanical disruption of the retina or eye. The syringe was removed and the eyelid was closed using sterile cotton-buds. The injection procedure was repeated daily until P5. To reduce damage, the eye was reopened at the line of the first cut and the syringe was inserted through the same hole in the sclera which could be identified by the visible hole in the retinal pigmented epithelium. About 1 mM epibatidine [epibatidine dihydrochloride hydrate, Sigma-Aldrich, E1145; dissolved in sterile saline (9 g NaCl per liter sterile water)] was injected daily from P0 to P5. Around 0.5 µL was injected at P0, increasing by 0.1 µL per day until 1 µL was given at P5. Control litters were injected with sterile physiological saline using the identical procedure. After surgery, animals were warmed until pink and moving normally before being returned to the home cage.

### Proportional Pixel Analysis

The ingrowth of fluorescent fibers to the dLGN and superficial gray layer of the SC was quantified by analyzing the intensity profiles of pixels within the dLGN or superficial gray layer of the SC on fluorescent microscope images taken on a Leica DMR fluorescence microscope with a DC500 camera and Leica Firecam software (Leica Microsystems, Milton Keynes, UK). Images from the control and manipulated hemisphere were taken with identical microscope and camera settings. In addition, image intensity was standardized between brains in ImageJ (Schneider et al. 2012) by measuring fluorescence intensity within ventral posterior-medial nucleus (VPM) on thalamic sections or periaqueductal gray on tectal sections. Any images, in which fluorescence intensity departed from the average was adjusted using the brightness tool in ImageJ.

The images acquired contralateral to the manipulated (removed/ injected) eye were compared with the images ipsilateral to the manipulated eye of the same brain (as an intra-animal control). Analysis was done blind to experimental condition. The region of interest for analysis was selected by the following criteria—the dLGN: the lateral border: “intralaminar” GFP or tdTomato bundle between dLGN and vLGN; ventral border: thick GFP/tdTomato-positive bundle between the dLGN and VPM; medial border: curved GFP/tdTomato bundle between the dLGN and the posterior thalamic nucleus (Po); the dorsal border: the edge between the dLGN tissue and the lateral ventricle. In the ventral border, the line selection tool was drawn through the mid-point of the GFP/tdTomato bundle. In the lateral corner of the dLGN: the dorsal, lateral, and ventral edges of the dLGN were defined as above. To outline just the lateral half of the dLGN, the “medial” outline was done halfway between the lateral edge of the nucleus and the medial edge of the nucleus. In the superficial gray layer: the ventral border: the border between the superficial gray layer and the optic nerve layer as assessed by the density of tdTomato-positive fibers (which are in thicker bundles in the optic nerve layer). The dorsal edge of the SC was used as the dorsal border of the superficial gray layer. The medial edge of the SC was used as the medial edge of the superficial gray layer. The lateral edge the superficial gray is where the dorsal edge and ventral edges meet.

In the selected area, the gray-scale intensity value of every pixel was captured using the Record Measurements tool in Adobe Photoshop CS5 (Adobe Systems Inc., CA, USA). This quantifies the number of pixels at every gray-scale intensity value (range 0–255). Using these data, the frequency of pixels above the threshold was calculated for every intensity threshold in the range, and normalized by the number of pixels within the area of the dLGN. This measures the proportion of pixels within the dLGN which are above any gray-scale intensity threshold.

Statistical comparisons were performed at every 10th pixel intensity threshold (10–120) for the thresholds above which the proportion of pixels drops from 1 and below which the proportion of pixels is above 0 in GraphPadPrism (GraphPad Software, CA, USA). Control and enucleated dLGN were compared using one-tailed, paired *t*-test. In all graphs, * = significant at *P* = 0.05, ** = significant at *P* = 0.005, *** = significant at *P* < 0.0005.

### Pixel Analysis Along a Line

Images were standardized for intensity as above, the same threshold intensity was used in each condition. A 300-µm long line was drawn from the dorsal edge of the dLGN to the ventral edge, midway along, and perpendicular to the dorsal edge. The plot profile tool in ImageJ was used to record the intensity value of pixels every 0.5 µm along this line.

### Carbocyanine Dye Tracing

Small crystals of DiI (1,1′-didodecyl-3,3,3′,3′-tetra-methylindocarbocyanine perchlorate, Invitrogen) were placed in the control and enucleated dLGN of fixed brains at P6/7, following monocular enucleation at P0. Brains were incubated in 0.05% PBSA at 37 °C for 5 weeks. Owing to scarcity of correctly placed DiI crystals, one brain from the Lpar1-eGFP line (Hoerder-Suabedissen and Molnár 2013) aged P7 was included in the analysis. All other brains were from the Golli-τ-eGFP line aged P6. Brains were embedded in 5% agarose and sectioned coronally to 50 µm on a vibrating microtome.

### Immunohistochemistry

Anti-GFP immunohistochemistry was performed on all Golli-τ-eGFP sections. Aggrecan immunohistochemistry was performed on coronal sections across the SC of the Rbp4-Cre::tdTomato mouse. VGluT1 and synaptophysin double immunohistochemistry was performed on dLGN-containing sections of enucleated Rbp4-Cre::tdTomato brains.

Sections for immunohistochemistry were blocked for 2 h at room temperature (RT), then incubated with primary antibody in blocking solution overnight at 4 °C. Incubation with secondary antibody in blocking solution was performed for 2 h at RT. Sections were counterstained with 5 µg/mL DAPI (dilactate, Invitrogen, D3571) for 5 min. Details of antibody combinations and block conditions are summarized in Table 1.

**Table 1:** Antibody combinations and block conditions used for immunohistochemistry

Strains	Primary antibodies	Secondary antibodies	Block
Golli-τ-eGFP	Rabbit anti-GFP 1 : 500 (Invitrogen, A11122)	Donkey anti-rabbit AlexaFluor488 1 : 500 (Invitrogen, A21206)	0.1% Triton X-100 (BDH, Poole, UK) 2% donkey serum (Sigma-Aldrich, Gillingham, UK) 0.1 M PBS
Rbp4-Cre::tdTomato	Mouse anti-synaptophysin (SY38) 1 : 100 (Abcam, ab8049) Guinea pig anti-VGluT1 1 : 10 000 (Chemicon, AB5905)	Donkey anti-mouse AlexaFluor488 1 : 500 (Invitrogen, A21202) Donkey anti-guinea pig biotin 1 : 100 (Jackson ImmunoResearch 706-065-148) followed by streptavidin-cy5 1 : 200 Jackson ImmunoResearch (016-170-084)	0.1% Triton X-100 2% donkey serum 0.1 M PBS
Rbp4-Cre::tdTomato Ntsr1-Cre::tdTomato	Mouse anti-chondroitin sulfate proteoglycan antibody, Core Protein Epitope, 1 : 2500 (Chemicon, MAB1581). Specific for aggrecan (Matthews et al. 2002; Brooks et al. 2013)	Donkey anti-mouse AlexaFluor488 1 : 500 (Invitrogen, A21202)	0.1% Triton X-100 2% donkey serum 0.1 M PBS

## Results

### Transgenic Mouse Lines That Specifically Label Layer V, Layer VI, and Layer VIa/VIb Corticothalamic Neurons

**Figure 1.**
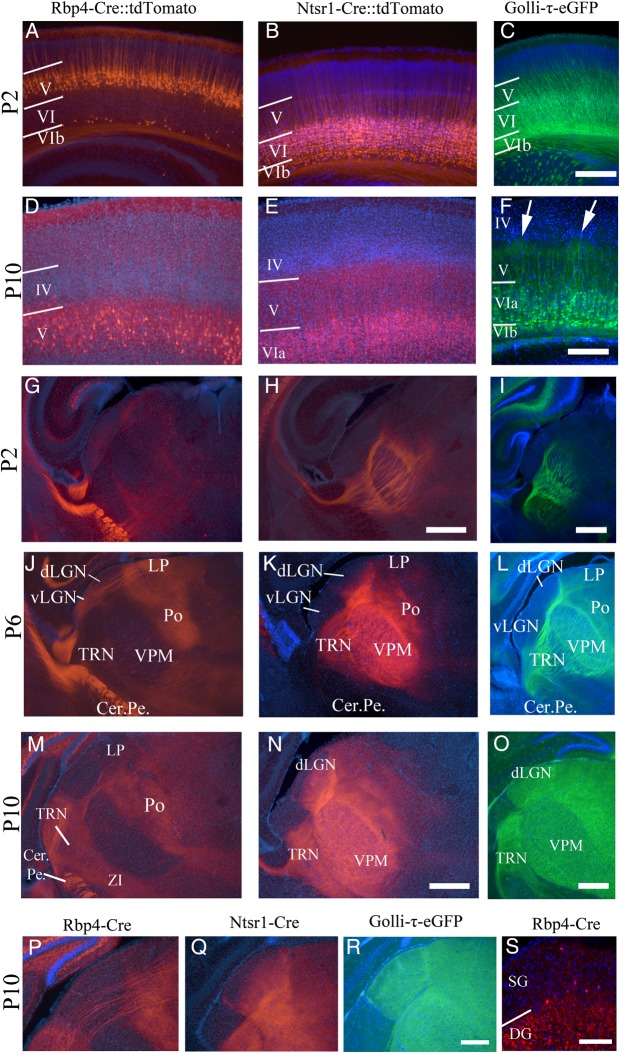
Fluorescent marker expression in the Rbp4-Cre::tdTomato, Ntsr1-Cre::tdTomato, and Golli-τ-eGFP mice. (*A*) Cre induced tdTomato expression in layer V pyramidal neurons of the Rbp4-Cre line at P2. (*B*) Cre-induced tdTomato expression is confined to layer VIa of the Ntsr1-Cre mouse at P2. (*C*) In the Golli-τ-eGFP mouse at P2, eGFP expression is in layer VI including sublayers VIa and VIb. (*D*) Rbp4-Cre::tdTomato layer V pyramidal neurons at P10. (*E*) Ntsr1-Cre::tdTomato layer VI neurons at P10. (*F*) Golli-τ-eGFP expressing VIa and VIb neurons at P10. Periphery-related barrel patterning is visible (white arrows) (*G*). Rbp4-Cre::tdTomato layer V corticothalamic fibers have entered the thalamus and project through the VPM and dLGN and project to the LP and Po at P2. They are also densely packed in the cerebral peduncle (*H*). Ntsr1-Cre::tdTomato layer VIa fibers are strongly visible in the TRN, VPM and reach Po at P2. (*I*) Golli-τ-eGFP fibers have reached the TRN and VPM at P2. (*J*) At P6, layer V tdTomato fibers have reached the medial, higher-order, thalamic nuclei while coursing through the first-order dLGN and VPM without barbarizations. Fibers remain absent from TRN. (*K*) At P6, the layer VIa tdTomato fibers are densely innervating the first-order VPM and are projecting into the dLGN, they have also started entering the most medial higher-order thalamic nuclei. (*L*) The layer VIa and VIb eGFP fibers are strongly visible in VPM and have reached more medial higher-order nuclei and have formed a line around the dLGN, but have not yet entered it at P6. (*M*) At P10, the layer V fibers innervate the LP and Po, but still do not branch in dLGN or VPM. Innervation of TRN and ZI has increased in density. (*N*) Ntsr1-Cre::tdTomato layer VI corticothalamic fibers arborize in the TRN, the dLGN, the VPM, the LP, and the Po. (*O*) Golli-τ-eGFP layer VI and VIb fibers arborize in the TRN, the dLGN, the VPM, the LP, and the Po. (*P*) Layer V tdTomato fibers run through the dLGN, but do not arborize in it. (*N*) Layer VI tdTomato fibers enter the dLGN from the ventral/medial edge and by P10 have not yet filled it. (*Q*) Layer VI and VIb fibers enter the dLGN and have filled it by P10. (*S*) Rbp4-Cre::tdTomato-positive layer V fibers innervate the superficial gray (SG) layer of the SC. Layer boundaries determined by DAPI. Images are representative of the *n* ≥ 3 brains per age and strain that were analyzed in detail. LP, lateral posterior thalamic nucleus; Po, posterior thalamic nucleus; dLGN, dorsal lateral geniculate nucleus; VPM, ventral posterior (medial) nucleus; TRN, thalamic reticular nucleus; Cer. Pe., cerebral peduncle; SG, superficial gray layer of SC; DG; deep gray layer of SC. Scale bars = *A*–*C* 125 µm, *D* and *E* 500 µm, *F* 500 µm, *G*–*I* 250 µm, *J* 50 µm.

Three cortical layers project to the thalamus, layer V, layer VIa, and layer VIb (called the subplate during development). We used 3 recently developed transgenic mouse lines to label pyramidal neurons in layer V, layer VIa separately, and layer VI (including VIa and VIb) unambiguously, in order to monitor the corticothalamic fibers as they grow into specific nuclei. The Rbp4-Cre mouse expresses Cre-recombinase in a subgroup of layer V pyramidal neurons and a small population of VIb neurons (GENSAT; Gong et al. 2007, 2003; Gerfen et al. 2013). When crossed with the STOP-floxed tdTomato Cre reporter line Ai14-R26R-tdTomato (Madisen et al. 2010), layer V pyramidal neurons and their dendrites and axons are labeled (Fig. 1*A*). Layer VIa neurons and neurites were labeled by crossing the Ntsr1-Cre mouse line, which expresses Cre-recombinase in layer VIa cortical neurons (GENSAT; Olsen et al. 2012; Gerfen et al. 2013), with the same tdTomato reporter line (Fig. 1*B*). The Golli-τ-eGFP mouse labels layer VI including both sublayers VIa and VIb as well as a small population of layer V cells (Jacobs et al. 2007; Piñon et al. 2009; Fig. 1*C*). We utilize these 3 patterns of labeling to distinguish the patterns of ingrowth of different cortical layers into the thalamus.

### Layer-Specific Corticothalamic Ingrowth to First-Order and Higher-Order Thalamic Nuclei

Layer V and layers VIa and VIb are integrated into distinct hierarchical thalamocortical relays in adulthood. Layer V fibers innervate exclusively higher-order thalamic nuclei; layer VIa and VIb fibers innervate both higher-order and first-order thalamic nuclei (Sherman and Guillery 1996, 1998, 2002). The transgenic lines used here demonstrate that this layer-specific innervation of thalamic nuclei is established as early as P2 (Fig. 1*G–I*). By P10, the layer V Rbp4-Cre::tdTomato-labeled fibers project through the first-order dLGN and VPM and arborize densely in the higher-order lateral posterior nucleus (LP) and Po (Fig. 1*M*). This is complementary to layer VIa and VIb fibers labeled by Ntsr1-Cre::tdTomato and Golli-τ-eGFP, which densely innervate the pre-thalamic TRN, the first-order VPM and dLGN, and the higher-order LP and Po (Fig. 1*H*,*I*).

### Rbp4-Cre::tdTomato-Positive Layer V Corticotectal Fibers

The Rbp4-Cre::tdTomato line also labels the layer V cortico-subcerebral projection neurons. Layer V cortico-subcerebral fibers are evident in the cerebral peduncle (Fig. 1*G*,*J*,*M*) and the SC (Fig. 1*S*). At P10, thick bundles are visible in the deep layers of the colliculus and de-fasciculated fibers are visible innervating the superficial gray layer of the SC (Fig. 1*S*). There are also sparse, faintly tdTomato-positive cells, primarily in the deeper layers of the SC (Figs 1*S* and 4; Supplementary Fig. 4).

### Monocular Enucleation in Neonates Causes Premature Entry of Layer VIa and VIb Fibers to the dLGN

To assess the role of retinal input on the ingrowth of corticothalamic fibers, we performed early retinal manipulations. Retinal input to the dLGN was removed by monocular enucleation at P0 in the Golli-τ-eGFP and Ntrs1-Cre::tdTomato mouse lines. Layer VIa and VIb fibers (Golli-τ-eGFP) or layer VIa fiber (Ntrs1-Cre::tdTomato) ingrowth to the dLGN were assessed over development. As expected, the cross-sectional area of the enucleated dLGN was reduced compared with control (see Supplementary Table 1; Cullen and Kaiserman-Abramof 1976; Heumann and Rabinowicz 1980).

**Figure 2.**
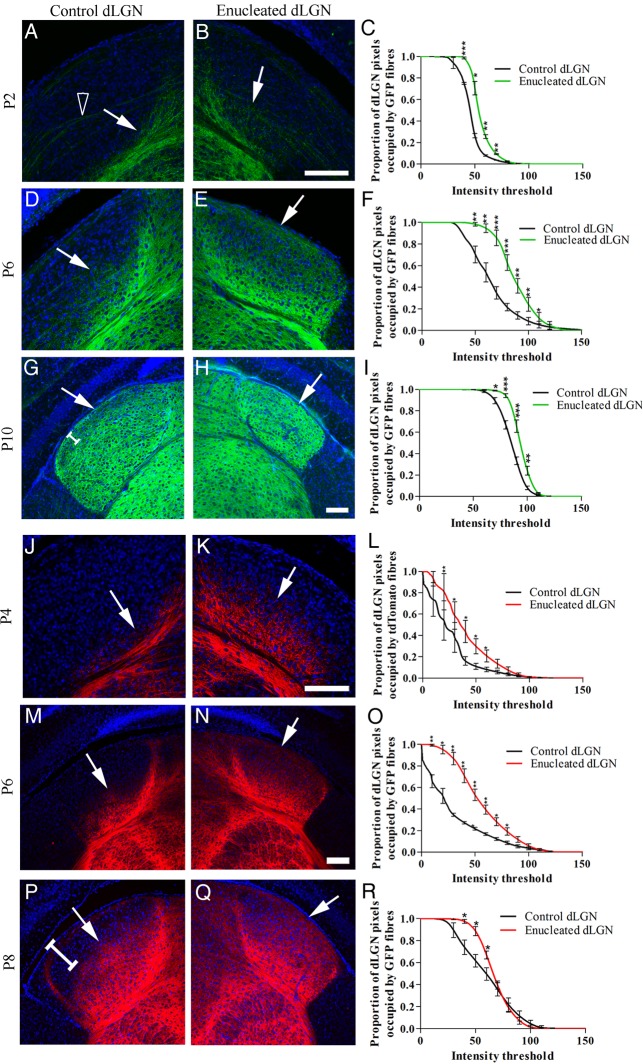
Fluorescently labeled layer VI and VIa corticothalamic fibers enter the dLGN prematurely after the removal of retinal input at birth by monocular enucleation. (*A*) Golli-τ-eGFP-positive VI and VIb fibers reach the ventral edge of the control dLGN by P2 (white arrow). Some eGFP-labeled fibers can be seen crossing the dLGN from the few layer V cells labeled (hollow arrow head). (*B*) By P2, Golli-τ-eGFP-positive VIa and VIb fibers begin to enter the enucleated dLGN (white arrow). (*C*) Proportionally more pixels are occupied by bright eGFP fibers in the enucleated dLGN compared with control dLGN, *n* = 3. (*D*) By P6, eGFP-positive fibers have entered the ventral half of the control dLGN (white arrow). (*E*) The eGFP fibers have reached the dorsal/lateral edge of the enucleated dLGN and extend throughout the nucleus (white arrow). (*F*) Proportionally more pixels are occupied by bright eGFP fibers in the enucleated dLGN compared with control dLGN, *n* = 6. (*G*,*H*) By P10, eGFP fibers have reached the dorsal/lateral edge of both the control and enucleated dLGN (white arrows); however, a less dense band remains in the control dLGN (white bracket). (*I*) Proportionally more pixels are occupied by bright eGFP fibers in the enucleated dLGN compared with control dLGN though the difference is less substantial, *n* = 5. (*J*) At P4, layer VI Ntsr1-Cre::tdTomato fibers have reached the ventral edge of the control dLGN, but do not yet enter (white arrow). (*K*) Layer VI Ntsr1-Cre::tdTomato fibers have entered the ventral half of the enucleated dLGN (white arrow). (*L*) Proportionally more pixels are occupied by bright tdTomato fibers in the enucleated dLGN compared with control, *n* = 3. (*M*) By P6, Ntsr1-Cre::tdTomato-positive layer VIa fibers have entered the ventral half of the control dLGN (white arrow). (*N*) Layer VIa tdTomato-labeled fibers have reached the dorsal/lateral edge of the enucleated dLGN (white arrow). (*O*) Proportionally more pixels are occupied by bright tdTomato fibers in the enucleated dLGN compared with control dLGN, the difference is greatest at this age, *n* = 4. (*P*) By P8, tdTomato fibers do not yet reach the dorsal/lateral edge of the control dLGN (white arrow and white bracket). (*Q*) The fibers are more densely arborized throughout the enucleated dLGN, but are not yet evenly distributed. (*R*) There are proportionally more pixels occupied by bright tdTomato-positive fibers in the control dLGN compared with the enucleated dLGN at P8, *n* = 4. Values on graphs are mean and standard error. Scale bars = 50 µm *A*–*E*, *G*–*H*, *I*–*J*, *M*–*Q*.

Following monocular enucleation, layer VIa and VIb fibers enter the enucleated dLGN prematurely (Fig. 2) from as early as P2 (Fig. 2*B*) and extend throughout the nucleus by P6 (Fig. 2*E*). In control brains, fibers enter dLGN at P6. Pixel intensity analysis on gray-scale images demonstrated a significantly higher proportion of pixels being occupied by bright eGFP fibers in enucleated compared with control dLGN at all ages studied (Fig. 2*C*,*F*,*I*). The Ntsr1-Cre::tdTomato-labeled layer VIa axons also enter the enucleated dLGN prematurely (Fig. 2*J–R*). A significantly higher proportion of pixels are occupied by bright tdTomato fibers in the enucleated compared with control dLGN (Fig. 2*L*,*O*,*R*). Using retrograde DiI tracing, we demonstrate that the majority of these fibers originate from layer VIa and VIb of the primary visual cortex (see Supplementary Fig. 2).

There was no difference in corticothalamic ingrowth to other thalamic nuclei (such as the visual higher-order nucleus LP) or the visual sector of the pre-thalamic TRN (see Supplementary Fig. 3).

### Intraocular Injections of Potent Cholinergic Agonist Epibatidine from Birth Causes Premature Entry of Layer VIa and VIb Golli-τ-eGFP Fibers to the dLGN

To differentiate between the effect of the loss of retinal axons and the loss of the coordinated retinal activity, we abolished retinal waves by intraocular injections of epibatidine. Epibatidine, a potent cholinergic agonist, de-sensitizes cholinergic receptors and thereby abolishes organized retinal waves and reduces retinal activity (Penn et al. 1998; Sun et al. 2008; Rebsam et al. 2009, 2012; Ackman et al. 2012). Epibatidine, or sterile saline control, was injected daily from P0 to P5. Golli-τ-eGFP layer VIa and VIb fiber ingrowth to the dLGN was analyzed at P6.

**Figure 3.**
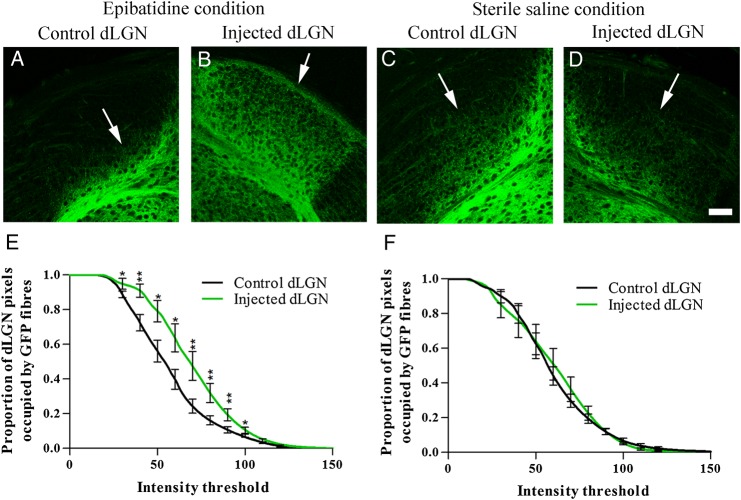
Layer VIa and VIb Golli-τ-eGFP fiber ingrowth to the dLGN following daily injections of epibatidine or sterile saline into one eye from birth until P6. (*A*) At P6, eGFP fibers have only entered the ventral edge of dLGN in the control (non-injected) side of epibatidine-treated animals. (*B*) eGFP VIa and VIb fibers have filled the epibatidine-treated dLGN at P6; *n* = 7. (*C* and *D*) Using sterile saline as a sham control, there is no difference between ingrowth in the control and saline-treated dLGN, with eGFP+ fibers only innervating the ventral half of dLGN; *n* = 8. (*E*) There are proportionally more pixels occupied by bright eGFP fibers in the epibatidine-treated dLGN to control dLGN. (*F*) There is no difference between control dLGN and sterile saline-treated dLGN. Values on graphs are mean and standard error. Scale bar = 25 µm.

Disruption of retinal waves by intraocular epibatidine caused premature entry of VIa and VIb eGFP-labeled fibers into dLGN (Fig. 3*A–E*). At P6, the VIa and VIb fibers have arborized throughout dLGN on the injected side only (Fig. 3*B*). eGFP-labeled fibers in control conditions (not injected or saline injected) are restricted to the ventral half of dLGN at P6 (Fig. 3*C–F*).

A higher proportion of pixels in the epibatidine-treated dLGN are occupied by eGFP fibers compared with the contralateral (control) dLGN (Fig. 3*E*). Epibatidine treatment reduced the cross-sectional area of dLGN compared with control (control area = 0.975 ± 0.13 mm^2^; epibatidine-treated area = 0.882 ± 0.12 mm^2^; mean ± SD; *P* < 0.05; see Supplementary Table 2). This size reduction was not as substantial as that observed following monocular enucleation. dLGN cross-sectional area was not significantly reduced following saline injections (control area = 1.445 ± 0.23 mm^2^; saline-treated area = 1.325 ± 0.31 mm^2^; mean ± SD; *P* > 0.1).

### Monocular Enucleation at Birth Causes Premature Entry of Layer V Fibers to the Superficial Gray Layer of the SC

The superficial gray layer of the SC also receives retinal input. We therefore asked whether layer V corticotectal fiber ingrowth to the superficial gray layer is also regulated by retinal input. To address this, we performed monocular enucleation on P0 Rbp4-Cre::tdTomato mice. Layer V fiber ingrowth to the superficial gray layer with and without retinal input was assessed over development.

**Figure 4.**
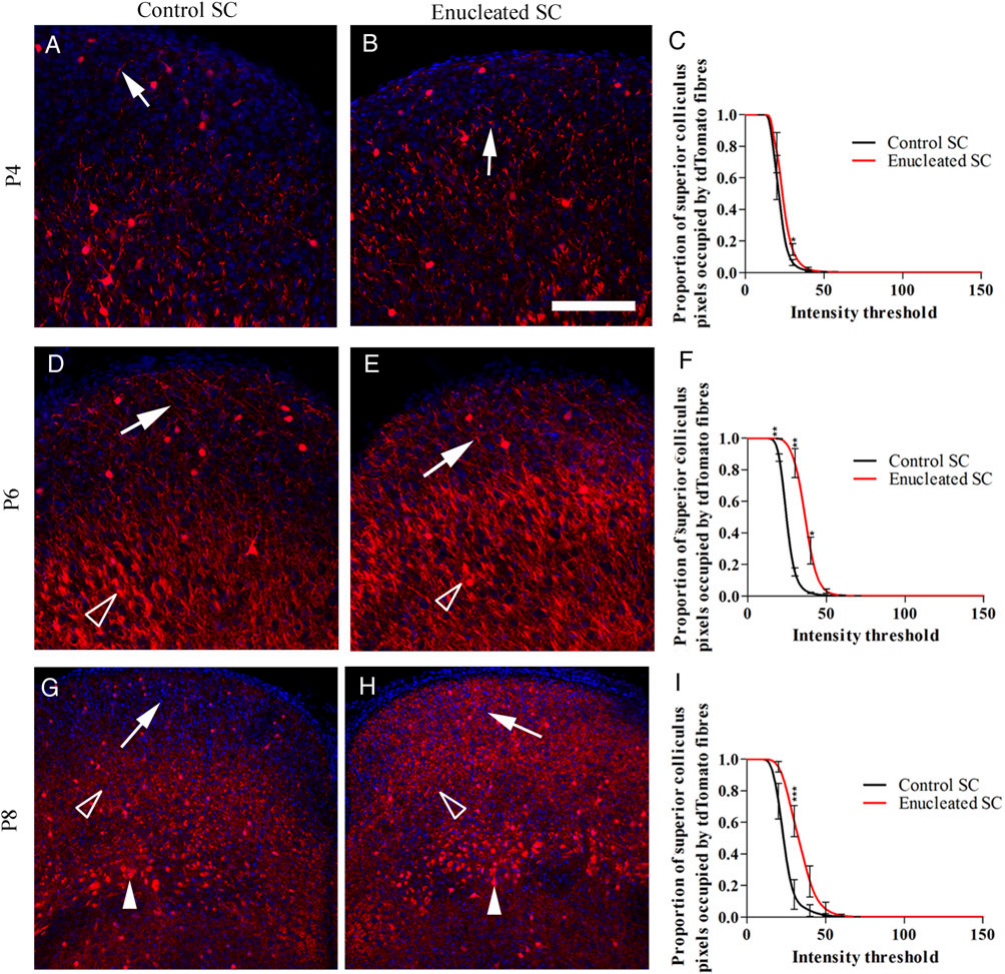
Premature entry of Rbp4-Cre::tdTomato-positive layer V fibers to the superficial gray layer of the SC following monocular enucleation at birth. (*A*) At P4, few layer V fibers are present in the superficial gray layer of the control SC (white arrow). (*B*) In the enucleated superficial gray layer, slightly more fibers are visible (white arrow). (*C*) At P4, there is no significant difference between the proportion of pixels occupied by tdTomato fibers in the superficial gray layer in the control or enucleated SC, *n* = 3. (*D*) At P6, fibers are visible in the control superficial gray layer (white arrow). Thick bundles of fibers are visible in the deeper layers (hollow arrow head). (*E*) Fibers are denser in the enucleated superficial gray layer. Thick bundles of fibers are visible in the deeper layers (hollow arrow head). (*F*) Proportionally more pixels are occupied by tdTomato fibers in the enucleated superficial gray compared with control, *n* = 4. (*G*) At P8, tdTomato-positive fibers are present in the control superficial gray layer (white arrow). At this age, the fibers appear to arborize in the optic nerve layer (hollow arrow head), but remain in bundles in the intermediate white layer (filled arrow head). (*H*) The layer V fibers densely fill the superficial gray layer of the enucleated SC (white arrow heads). Fibers are visible arborizing in the optic nerve layer (hollow arrow head) and running through the intermediate white layer in thicker bundles (filled arrow head). (*I*) Proportionally more pixels are occupied by tdTomato fibers in the enucleated superficial gray compared with control, *n* = 3. Values on graphs are mean and standard error. Scale bar = 50 µm.

Monocular enucleation caused premature ingrowth of tdTomato-labeled layer V fibers to the superficial gray layer (Fig. 4). At P4, qualitatively more fibers are visible in the superficial gray layer that lacks retinal input (Fig. 4*A*,*B*), but there is no difference in the proportion of pixels occupied by bright tdTomato fibers (Fig. 4*C*). At P6 and P8, the layer V fibers are considerably denser in the enucleated superficial gray layer than the control side (Fig. 4*D*,*E*,*G*,*H*). Significantly more pixels are occupied by bright tdTomato fibers in the superficial gray layer without retinal input, compared with the control (Fig. 4*F*,*I*). There is no apparent difference in other layers of the SC.

Previous research has demonstrated that aggrecan, a repulsive chondroitin sulfate proteoglycan, may be partially responsible for the specific temporal regulation of corticothalamic ingrowth to the dLGN (Brooks et al. 2013). We therefore performed aggrecan immunohistochemistry on the superficial gray layer of the SC. No significant difference in aggrecan labeling in the superficial gray layer of the superior colliculus was found between enucleated and control side (*n* = 3 brains). Moreover, layer V Rbp4-Cre::tdTomato-positive fibers densely innervate regions which express aggrecan (see Supplementary Fig. 4).

### Monocular Enucleation at Birth Causes Cross-Hierarchical Rewiring of Layer V Fibers to Aberrantly Innervate the First-Order dLGN

Next, we tested whether the specific ingrowth of layer V fibers to higher-order thalamic nuclei is altered to include first-order nuclei following loss of retinal input.

**Figure 5.**
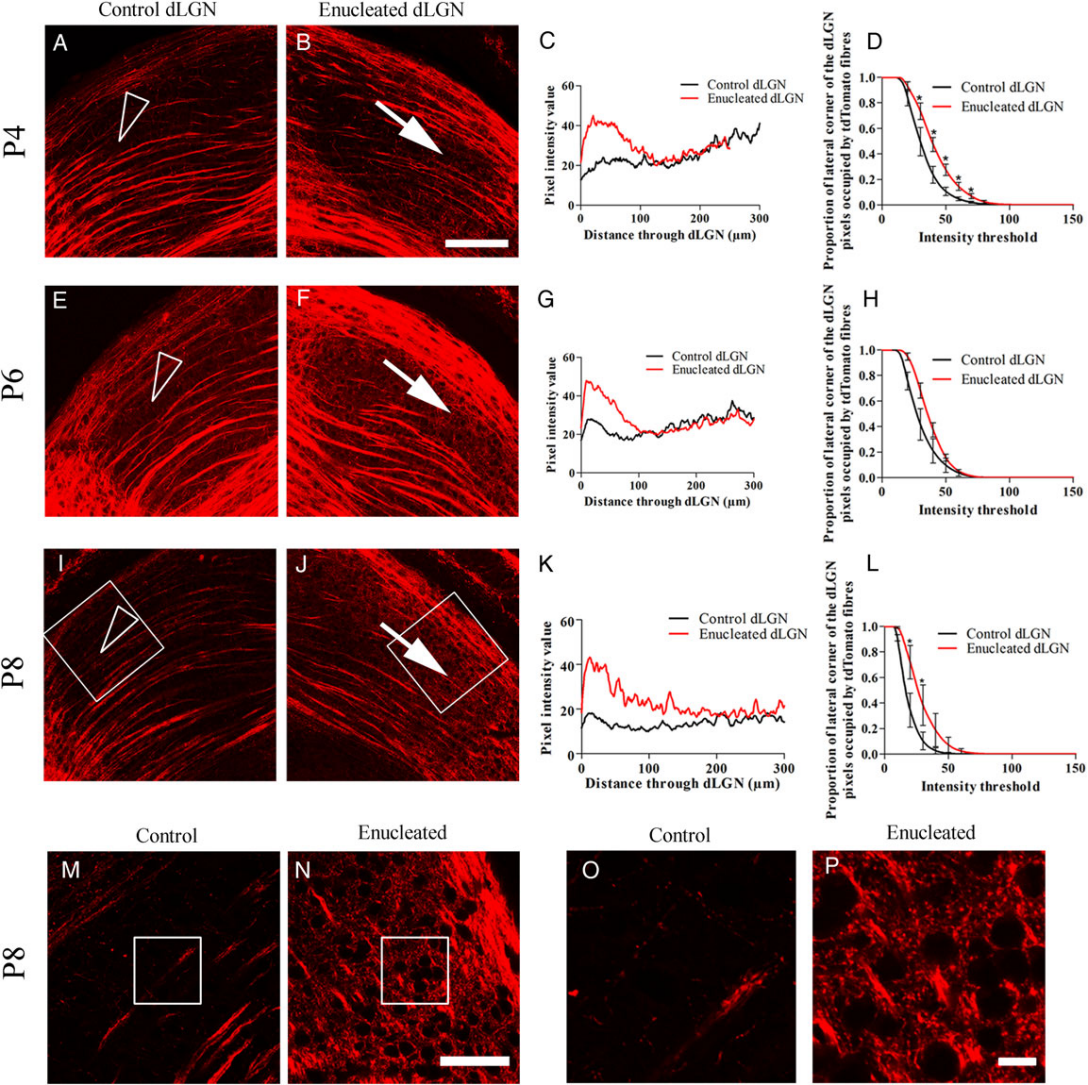
Rbp4-Cre::tdTomato layer V fibers rewire to innervate the dLGN after monocular enucleation at birth. (*A*) At P4, layer V Rbp4-Cre::tdTomato fibers cross the control dLGN without arborizing (arrow head). (*B*) In the dLGN-lacking retinal input the layer V fibers continue to cross dLGN. However, fibers appear denser near the dorsal edge and also appear to arborize in the lateral corner (white arrow). (*C*) The brightness of pixels along a line from the dorsal/lateral edge of the dLGN shows brighter pixels in the dorsal region of the dLGN-lacking retinal input compared with control. (*D*) Proportionally more pixels are occupied by brighter tdTomato fibers in the lateral corner of the dLGN-lacking retinal input compared with the control dLGN, *n* = 3. (*E–H*) At P6, layer V tdTomato fibers are denser in a dorsal band of the dLGN without retinal input and arborize in the lateral corner of the dLGN-lacking retinal input (white arrow) unlike the control dLGN which they grow through (arrow head), *n* = 4. (*I–L*) At P8, in the control dLGN, the fibers are sparse and grow through without arborizing (arrow head). In the dLGN-lacking retinal input, the layer V fibers are dense near the dorsal edge and arborize in the lateral corner (white arrow), *n* = 3. (*M* and *N*) High power images of the area demarcated by the white box in *I* and *J*, demonstrating sparse labeling in the control dLGN (*M*) compared with dense tdTomato fibers in the dLGN-lacking retinal input (*N*). (*O* and *P*) Enlarged image of area outlined in *M* and *N*, respectively, to show clear small fibers surrounding cells in the enucleated dLGN, which are not present in the control dLGN. Values on graphs are mean ± standard error. Scale bar = 50 µm *A*–*J*, 100 µm *M* and *N*, 10 µm *O* and *P*.

During normal development, layer V Rbp4::tdTomato-positive fibers project through the dLGN without arborizing (Fig. 5*A*,*E*,*I*). Following enucleation, tdTomato-labeled fibers arborize in parts of the enucleated dLGN. At P2, there is a thickening of the layer V tdTomato-positive fiber bundles at the dorsal/lateral edge of the dLGN (Fig. 5*B*). This is also evident at P6 and P8 (Fig. 5*F*,*J*). Furthermore, in the lateral dLGN, there is branching of thin fibers from the thick layer V bundles at all ages investigated (Fig. 5*B*,*F*,*J—*white arrows). The pixel intensity along a line from the dorsal/lateral edge of the dLGN (the border adjacent to the ventricle) to the ventral/medial edge of the dLGN is higher in the most dorsal 100 µm of the enucleated dLGN compared with control (Fig. 5*C*,*G*,*K*). Proportional pixel analysis of the lateral corner of the dLGN showed a significant increase in area occupied by high intensity pixels in the enucleated dLGN at P4 and P8 (Fig. 5*D*,*H*,*L*).

**Figure 6.**
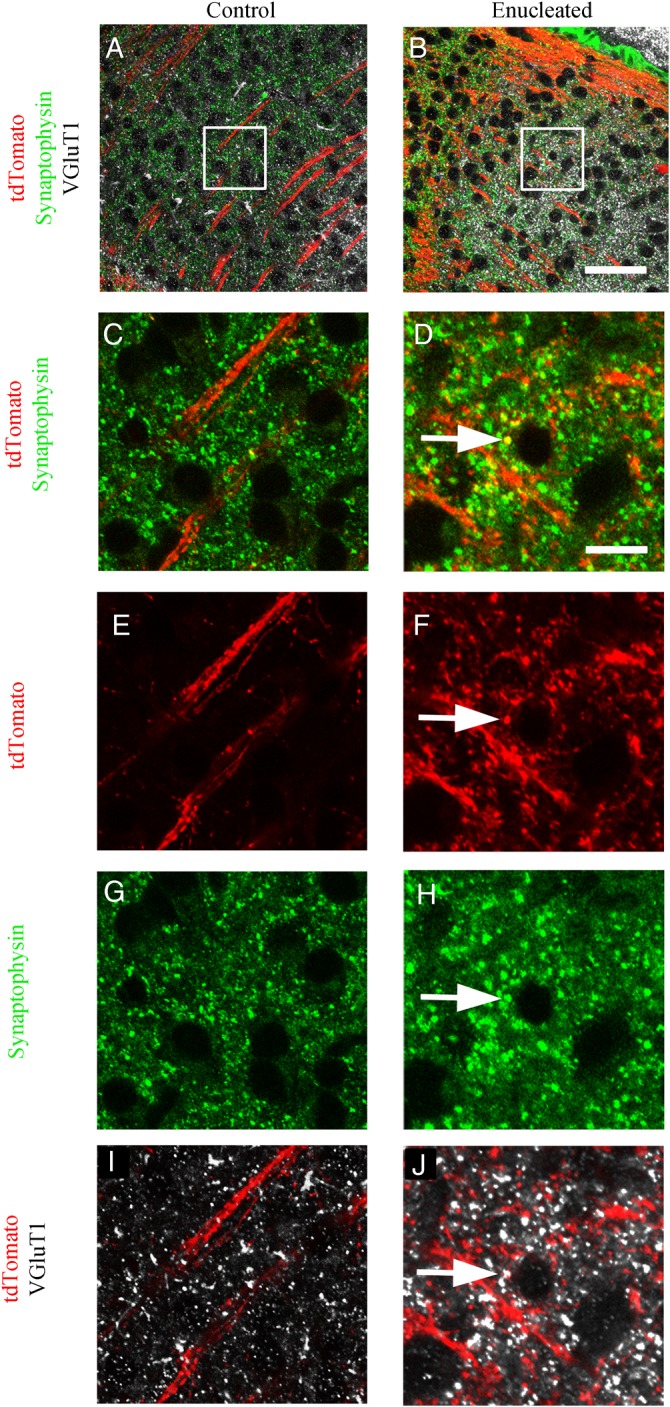
VGluT1 and synaptophysin immunohistochemistry on Rbp4-Cre::tdTomato demonstrate colocalization of tdTomato with cortical synapses. (*A* and *B*) VGluT1 labels cortical synapses in white, and synaptophysin in green labels all synapses. Both are visible in the control and enucleated dLGN, but VGluT1 labeling is denser and extends more dorsally into dLGN (*C*). In the control dLGN, layer V tdTomato fibers course through the dLGN but do not produce structures that overlap with synaptophysin labeled synapses. (*D*) In the enucleated dLGN, the red fibers include structures that are labeled with synaptophysin, suggesting the fibers terminate in synapses. (*E*) In the control dLGN, there are very few red structures corresponding to the tdTomato-positive layer V fibers coursing through the dLGN and there are no tdTomato synapses colabeled with synaptophysin (*G*) or VGluT1 (*I*). (*F*) In the enucleated dLGN, many fine red fibers arise from the red fibers traversing dLGN, and small round swellings are visible which are triple-labeled with tdTomato, synaptophysin (*H*), and VGluT1 (*J*). VGluT1 labels cortical synapses, which suggests that the tdTomato swellings are cortical synapses from layer V fibers. *n* = 3. Scale bar *A* and *B* = 50 µm, *C*–*J* = 5 µm.

To assess whether the layer V tdTomato-positive fibers in the dLGN are forming synapses, we performed VGluT1 and synaptophysin double immunohistochemistry. VGluT1 labels cortically derived glutamatergic synapses, whereas synaptophysin is located at all presynaptic terminals. VGlut1 and synaptophysin labeling is more dense in the enucleated dLGN than in the control dLGN. On the enucleated side, some Rbp4-Cre::tdTomato-labeled swellings colocalize with VGluT1 and synaptophysin, indicating that the small-side branches present exclusively on the enucleated side are also forming synapses. Such triple-labeled structures were not observed on the control dLGN side, in which the Rbp4-Cre::tdTomato-labeled fibers form tight bundles with hardly any side branches (Fig. 6).

A recent study demonstrated that a driver-like input from cells in the superficial gray layer of the SC innervates the dLGN (Bickford et al. 2015). There are sparse Rbp4-cre::tdTomato-positive cells, in the SC (Fig. 1*S*), but they are primarily located in the deeper layers of SC. Moreover, the tdTomato-positive cells in SC are only faintly labeled with tdTomato in comparison with the cells in cortical layer V and the fibers visible in dLGN. They are therefore unlikely to produce the dense and strongly labeled fibers seen in the dLGN following enucleation. Lastly, some of the tdTomato-positive fibers in the enucleated dLGN colocalize with VGluT1, which labels cortical synapses (Ni et al. 1995; Varoqui et al. 2002). In adult mouse, the cells in the superficial gray layer of the SC express only VGluT2 mRNA, not VGluT1 (Lein et al. 2007). We therefore suggest that the tdTomato-positive fibers in the dLGN following enucleation in the Rbp4-cre::tdTomato mouse are cross-hierarchical rewired fibers from layer V of the cortex.

## Discussion

### Layer-Specific Ingrowth to Thalamic Nuclei Based on the Thalamic Relay Hierarchy

Here, we demonstrate that the ingrowth of cortical fibers to the thalamus is highly nucleus specific as early as P2. Distinct corticofugal subpopulations initially project to the thalamic nuclei which they will innervate in adulthood. This pattern is delineated by the hierarchies of inputs into which the thalamic nuclei integrate (first order or higher order). As early as P2, layer V fibers exclusively target higher-order thalamic nuclei; merely coursing through the more lateral first-order nuclei. Layer VIa fibers on the other hand innervate both, entering the lateral first-order nuclei early and the higher-order nuclei days later.

This early patterning suggests that intrinsic guidance mechanisms distinguishing first-order from higher-order pathways within the thalamic nuclei may be specified early unlike other parts of the developing nervous systems, which rely on synaptic pruning. Guidance cues, which distinguish first- and higher-order nuclei, may direct V and VIa fibers to their appropriate target, or the different corticothalamic subpopulations may be differentially responsive to guidance mechanisms. The specific gene expression patterns of subclasses of cortical projection neurons is well understood (Molyneaux et al. 2007; Greig et al. 2013), but to date cues determining the guidance of corticothalamic fibers to the different thalamic nuclei have not been elucidated on a large scale. Recent work by Brooks et al. (2013) identified aggrecan as a guidance molecule which contributes to the timing of layer VI ingrowth into dLGN.

### Retinal Input Regulates Cortical Ingrowth to Subcortical Targets That are Jointly Innervated by the Retina and the Cortex

Layer VIa and VIb corticothalamic fibers, which innervate the dLGN, grow into it several days after birth (Grant et al. 2012). Layer V fibers, which innervate the superficial gray layer of the SC also enter the superficial gray layer several days after birth. Retinal fibers innervate both dLGN and the superficial gray layer of SC, and do so days before either population of cortical fibers innervates the same structure. In fact, retinal fibers have mature synapses capable of transmitting spontaneous waves of activity from the retina to the dLGN at birth (Mooney et al. 1996; Ackman et al. 2012).

Here, we present results showing that for 2 distinct visual system structures (the dLGN and the superficial gray layer of the SC), cortical fibers enter the targets that they share with the retinal fibers prematurely in the absence of retinal input.

Our results extend previous conclusions drawn from Golli-τ-eGFP-positive fiber ingrowth into dLGN in the math5^−/−^ mutant, which lacks retinal ganglion cells (Brooks et al. 2013; Seabrook et al. 2013), and following enucleation in the Golli-τ-eGFP mouse (Brooks et al. 2013). Taken together, these results demonstrate that retinal input regulates and delays the ingrowth of cortical fibers into targets shared by both.

To distinguish between the role of the retinal axons (absent after enucleation and in the math5^−/−^ mutant) and the role of the retinal activity transmitted by them, we performed intraocular injections of epibatidine to disrupt early spontaneous retinal activity. Our results demonstrated that the loss of patterned retinal activity (without loss of axons) also causes premature ingrowth of layer VIa and VIb fibers to dLGN. These results do not distinguish whether the effect is due to the substantial reduction in retinal activity or due to the loss of retinal patterning following epibatidine injections. We observed an increase in the proportion of pixels occupied by bright fluorescently labeled fibers in both experimental manipulations. The difference between enucleated/epibatidine-treated dLGN and control is unlikely to be due to the reduced volume of treated dLGN because the analysis measures distribution of density rather than area occupied by fibers. Furthermore, the fibers, especially at P6, clearly extend further into the dLGN from the medial border in absolute distance. Epibatidine injections did cause a decrease in the volume of the dLGN, indicating that there may be some retinal damage, which may have additionally impacted on corticothalamic ingrowth. To address this, it would be informative to assess ingrowth of Golli-τ-eGFP-positive VIa and VIb fibers on a genetic background in which the retinal waves are disrupted such as the nicotinic cholinergic receptor β2 subunit knockout mouse (Bansal et al. 2000; Rossi et al. 2001; Stafford et al. 2009).

Aggrecan has been shown to act as a timing molecule in delaying corticothalamic ingrowth to the dLGN for several days after birth perhaps in response to retinal input (Brooks et al. 2013). Our epibatidine injection experiments in combination with Brooks and colleagues' results suggest that the loss of transmission of retinal activity to the dLGN by monocular enucleation and epibatidine injections may cause altered expression of molecular cues including aggrecan which contribute to the temporal regulation of layer VIa and VIb ingrowth into the dLGN.

### A Common Mechanism for Cortical Guidance Preventing Early Ingrowth to Retinal Target Tissue

Our results address 2 systems, which receive input from both the retina and the cortex. Both in the retino-geniculo-cortical system and in the extrageniculo-cortical (retina–colliculus–LP–cortex) system, the retinal fibers innervate their target prior to cortical ingrowth. In both systems, the loss of retinal input causes premature ingrowth of cortical fibers to the target. This suggests that there may be a common mechanism by which retinal fibers prevent cortical fibers from entering their joint target.

Given the role of aggrecan in the premature ingrowth of cortical fibers to dLGN after the loss of retinal input (Brooks et al. 2013), we tested whether aggrecan could also be involved in premature entry of layer V fibers to the superficial gray layer of the SC. Our results demonstrated that aggrecan labeling in the superficial gray layer of the SC was not significantly affected by monocular enucleation and that, in the SC, layer V Rbp4-Cre::tdTomato-positive fibers densely innervate deeper layers which express aggrecan. These results suggest that there may be different molecular mechanisms by which retinal fibers prevent different populations of cortical fibers from entering the dLGN and the SC. This is consistent with the result that layer V corticothalamic fibers are not repelled by aggrecan expression in the higher-order thalamic nuclei either.

### Does Premature Entry of Corticothalamic Layer VIa and VIb Fibers Disrupt the Retinogeniculate Circuit?

From P7 to P9, retinal input to the mouse lateral geniculate can occupy as much as 50% of the surface area of dLGN relay neurons, including both proximal and distal regions of their dendrites (Ziburkus and Guido 2006; Guido 2008). This early extensive input is then refined by eye-specific segregation and synaptic pruning, leaving each thalamic relay cell to be innervated by 1–3 retinal ganglion cells by 2 weeks after birth (Godement et al. 1984; Chen and Regehr 2000; Jaubert-Miazza et al. 2005). Thus, during the earliest postnatal weeks, the layer VIa and VIb fibers and the retinal fibers are directly competing for contact surface area on the dLGN relay neurons.

We hypothesize that retinal fibers prevent early entry of corticothalamic fibers to the dLGN or SC, ensuring that appropriate synapse sites are available for the retinal fibers. As such, whether premature ingrowth of layer VIa corticothalamic fibers by itself can disrupt normal ingrowth of the retinogeniculate system will be important to establish.

It will also be interesting to expand this question to assess whether the geniculo-cortical pathway is heavily altered by the inappropriate corticothalamic connections and the missing retinal inputs produced by the enucleations; and, if so, whether compensatory cortical plasticity allows a relatively normal functional circuit.

### Loss of Retinal Input Enables Cross-Hierarchical Rewiring of Layer V Corticothalamic Inputs to the dLGN

Our results demonstrate that removal of retinal input allows layer V corticothalamic fibers to arborize and synapse within the first-order dLGN. To our knowledge, these are some of the first results that suggest there is cross-hierarchical corticothalamic plasticity after monocular enucleation. Cross-hierarchical rewiring has been previously demonstrated in the thalamocortical system (Pouchelon et al. 2014).

This cross-hierarchical rewiring may occur because the loss of retinal driver input to the dLGN causes the dLGN to appear more similar to a higher-order thalamic nucleus. In this case, one might predict that during normal development, the early dLGN may be similar to the LP. Alternatively, the loss of retinal driver input may increase available synaptic sites in the dLGN with reduced fiber competition. These results concur with early electron microscopy results which demonstrate that after enucleation, the synaptic sites which normally receive retinal input become innervated by large terminals with round synaptic vesicles (characteristics of driver terminals; Cullen and Kaiserman-Abramof 1976). Furthermore, the region which is innervated by the re-wired fibers coincides with the dorsolateral “shell” of the dLGN which normally receives only contralateral input (no ipsilateral contribution) and therefore lacks all driver input after monocular enucleation (Reese 1988; Grubb and Thompson 2004).

In this study, we emphasize the role of sensory input on the timing and geography of corticothalamic ingrowth by demonstrating premature ingrowth and cross-hierarchical rewiring of corticofugal connectivity after sensory deprivation. The contribution of such rewiring to plasticity and re-specification of thalamic projection neurons should be further considered.

## Supplementary Material

Supplementary material can be found at http://www.cercor.oxfordjournals.org/online.

## Funding

Our laboratory is supported by MRC (G00900901), BBSRC (BB/I021833/1), and The Wellcome Trust (092071/Z/10/Z). Funding to pay the Open Access publication charges for this article was provided by RCUK Open Access Block Grant for University of Oxford.

## Supplementary Material

Supplementary Data

## References

[BHV315C1] Ackman JB, Burbridge TJ, Crair MC 2012 Retinal waves coordinate patterned activity throughout the developing visual system. Nature. 490:219–225. 23060192 10.1038/nature11529 PMC3962269

[BHV315C2] Bansal A, Singer JH, Hwang BJ, Xu W, Beaudet A, Feller MB 2000 Mice lacking specific nicotinic acetylcholine receptor subunits exhibit dramatically altered spontaneous activity patterns and reveal a limited role for retinal waves in forming ON and OFF circuits in the inner retina. J Neurosci. 20:7672–7681. 11027228 10.1523/JNEUROSCI.20-20-07672.2000 PMC6772851

[BHV315C3] Bickford ME, Zhou N, Krahe TW, Govindaiah G, Guido W 2015 Retinal and tectal “driver-like” inputs converge in the shell of the mouse dorsal lateral geniculate nucleus. J Neurosci. 35 (29):10523–10534. 26203147 10.1523/JNEUROSCI.3375-14.2015 PMC4510292

[BHV315C4] Brooks JM, Su J, Levy C, Wang JS, Seabrook TA, Guido W, Fox MA 2013 A molecular mechanism regulating the timing of corticogeniculate innervation. Cell Rep. 5:573–581. 24183669 10.1016/j.celrep.2013.09.041 PMC3849812

[BHV315C5] Chen C, Regehr WG 2000 Developmental remodeling of the retinogeniculate synapse. Neuron. 28:955–966. 11163279 10.1016/s0896-6273(00)00166-5

[BHV315C6] Cullen MJ, Kaiserman-Abramof IR 1976 Cytological organization of the dorsal lateral geniculate nuclei in mutant anophthalmic and postnatally enucleated mice. J Neurocytol. 5:407–424. 993820 10.1007/BF01181648

[BHV315C7] Deschênes M, Bourassa J, Pinault D 1994 Corticothalamic projections from layer V cells in rat are collaterals of long-range corticofugal axons. Brain Res. 664:215–219. 7895031 10.1016/0006-8993(94)91974-7

[BHV315C8] Drager UC, Hubel DH 1975 Responses to visual stimulation and relationship between visual, auditory, and somatosensory inputs in mouse superior colliculus. J Neurophysiol. 38:690–713. 1127462 10.1152/jn.1975.38.3.690

[BHV315C9] Gensat. http://www.gensat.org/cre.jsp 2013.

[BHV315C10] Gerfen CR, Paletzki R, Heintz N 2013 GENSAT BAC Cre-recombinase driver lines to study the functional organization of cerebral cortical and basal ganglia circuits. Neuron. 80:1368–1383. 24360541 10.1016/j.neuron.2013.10.016 PMC3872013

[BHV315C11] Godement P, Salaun J, Imbert M 1984 Prenatal and postnatal development of retinogeniculate and retinocollicular projections in the mouse. J Comp Neurol. 230:552–575. 6520251 10.1002/cne.902300406

[BHV315C12] Gong S, Doughty M, Harbaugh CR, Cummins A, Hatten ME, Heintz N, Gerfen CR 2007 Targeting Cre recombinase to specific neuron populations with bacterial artificial chromosome constructs. J Neurosci. 27:9817–9823. 17855595 10.1523/JNEUROSCI.2707-07.2007 PMC6672645

[BHV315C13] Gong S, Zheng C, Doughty ML, Losos K, Didkovsky N, Schambra UB, Nowak NJ, Joyner A, Leblanc G, Hatten ME et al 2003 A gene expression atlas of the central nervous system based on bacterial artificial chromosomes. Nature. 425:917–925. 14586460 10.1038/nature02033

[BHV315C14] Grant EL, Hoerder-Suabedissen A, Molnár Z 2012 Development of the corticothalamic projections. Front Neurosci. 6 14:1–14. 22586359 10.3389/fnins.2012.00053 PMC3343305

[BHV315C15] Greig LC, Woodworth MB, Galazo MJ, Padmanabhan H, Macklis JD 2013 Molecular logic of neocortical projection neuron specification, development and diversity. Nat Rev Neurosci. 14:755–769. 24105342 10.1038/nrn3586 PMC3876965

[BHV315C16] Grubb MS, Thompson ID 2004 Biochemical and anatomical subdivision of the dorsal lateral geniculate nucleus in normal mice and in mice lacking the beta2 subunit of the nicotinic acetylcholine receptor. Vision Res. 44:3365–3376. 15536004 10.1016/j.visres.2004.09.003

[BHV315C17] Guido W 2008 Refinement of the retinogeniculate pathway. J Physiol. 586:4357–4362. 18556365 10.1113/jphysiol.2008.157115 PMC2614014

[BHV315C18] Heumann D, Rabinowicz T 1980 Postnatal development of the dorsal lateral geniculate nucleus in the normal and enucleated albino mouse. Exp Brain Res. 38:75–85. 7351229 10.1007/BF00237933

[BHV315C19] Hoerder-Suabedissen A, Molnár Z 2013 Molecular diversity of early-born subplate neurons. Cereb Cortex. 23:1473–1483. 22628460 10.1093/cercor/bhs137

[BHV315C20] Hooks BM, Chen C 2006 Distinct roles for spontaneous and visual activity in remodeling of the retinogeniculate synapse. Neuron. 52:281–291. 17046691 10.1016/j.neuron.2006.07.007

[BHV315C21] Jacobs EC, Campagnoni C, Kampf K, Reyes SD, Kalra V, Handley V, Xie YY, Hong-Hu Y, Spreur V, Fisher RS et al 2007 Visualization of corticofugal projections during early cortical development in a tau-GFP-transgenic mouse. Eur J Neurosci. 25:17–30. 17241263 10.1111/j.1460-9568.2006.05258.x

[BHV315C22] Jaubert-Miazza L, Green E, Lo FS, Bui K, Mills J, Guido W 2005 Structural and functional composition of the developing retinogeniculate pathway in the mouse. Vis Neurosci. 22:661–676. 16332277 10.1017/S0952523805225154

[BHV315C23] Jones EG 2002 Thalamic circuitry and thalamocortical synchrony. Philos Trans Roy Soc Lond Ser B Biol Sci. 357:1659–1673. 12626002 10.1098/rstb.2002.1168 PMC1693077

[BHV315C24] Jones EG 2001 The thalamic matrix and thalamocortical synchrony. Trends Neurosci. 24:595–601. 11576674 10.1016/s0166-2236(00)01922-6

[BHV315C25] Lam YW, Sherman SM 2013 Activation of both Group I and Group II metabotropic glutamatergic receptors suppress retinogeniculate transmission. Neuroscience. 242:78–84. 23558090 10.1016/j.neuroscience.2013.03.043 PMC3654074

[BHV315C26] Lein ES, Hawrylycz MJ, Ao N, Ayres M, Bensinger A, Bernard A, Boe AF, Boguski MS, Brockway KS, Byrnes EJ et al 2007 Genome-wide atlas of gene expression in the adult mouse brain. Nature. 445:168–176. 17151600 10.1038/nature05453

[BHV315C27] Madisen L, Zwingman TA, Sunkin SM, Oh SW, Zariwala HA, Gu H, Ng LL, Palmiter RD, Hawrylycz MJ, Jones AR et al 2010 A robust and high-throughput Cre reporting and characterization system for the whole mouse brain. Nat Neurosci. 13:133–140. 20023653 10.1038/nn.2467 PMC2840225

[BHV315C28] Matthews RT, Kelly GM, Zerillo CA, Gray G, Tiemeyer M, Hockfield S 2002 Aggrecan glycoforms contribute to the molecular heterogeneity of perineuronal nets. J Neurosci. 22:7536–7547. 12196577 10.1523/JNEUROSCI.22-17-07536.2002 PMC6757962

[BHV315C29] Molyneaux BJ, Arlotta P, Menezes JR, Macklis JD 2007 Neuronal subtype specification in the cerebral cortex. Nat Rev Neurosci. 8:427–437. 17514196 10.1038/nrn2151

[BHV315C30] Mooney R, Penn AA, Gallego R, Shatz CJ 1996 Thalamic relay of spontaneous retinal activity prior to vision. Neuron. 17:863–874. 8938119 10.1016/s0896-6273(00)80218-4

[BHV315C31] Ni B, Wu X, Yan G-M, Wang J, Paul SM 1995 Regional expression and cellular localization of the Na^+^-dependent inorganic phosphate cotransporter of rat brain. J Neurosci. 15:5789–5799. 7643219 10.1523/JNEUROSCI.15-08-05789.1995 PMC6577628

[BHV315C32] Olsen SR, Bortone DS, Adesnik H, Scanziani M 2012 Gain control by layer six in cortical circuits of vision. Nature. 483:47–52. 22367547 10.1038/nature10835 PMC3636977

[BHV315C33] Penn AA, Riquelme PA, Feller MB, Shatz CJ 1998 Competition in retinogeniculate patterning driven by spontaneous activity. Science. 279:2108–2112. 9516112 10.1126/science.279.5359.2108

[BHV315C34] Piñon MC, Jethwa A, Jacobs E, Campagnoni A, Molnár Z 2009 Dynamic integration of subplate neurons into the cortical barrel field circuitry during postnatal development in the Golli-tau-eGFP (GTE) mouse. J Physiol. 587:1903–1915. 19289548 10.1113/jphysiol.2008.167767 PMC2689332

[BHV315C35] Pouchelon G, Gambino F, Bellone C, Telley L, Vitali I, Luscher C, Holtmaat A, Jabaudon D 2014 Modality-specific thalamocortical inputs instruct the identity of postsynaptic L4 neurons. Nature. 511 (7510):471–474. 24828045 10.1038/nature13390

[BHV315C36] Rebsam A, Bhansali P, Mason CA 2012 Eye-specific projections of retinogeniculate axons are altered in albino mice. J Neurosci. 32:4821–4826. 22492037 10.1523/JNEUROSCI.5050-11.2012 PMC3329942

[BHV315C37] Rebsam A, Petros TJ, Mason CA 2009 Switching retinogeniculate axon laterality leads to normal targeting but abnormal eye-specific segregation that is activity dependent. J Neurosci. 29:14855–14863. 19940181 10.1523/JNEUROSCI.3462-09.2009 PMC2829946

[BHV315C38] Reese BE 1988 “Hidden lamination” in the dorsal lateral geniculate nucleus: the functional organization of this thalamic region in the rat. Brain Res Rev. 13:119–137. 10.1016/0165-0173(88)90017-3 3289687

[BHV315C39] Rossi FM, Pizzorusso T, Porciatti V, Marubio LM, Maffei L, Changeux JP 2001 Requirement of the nicotinic acetylcholine receptor beta 2 subunit for the anatomical and functional development of the visual system. Proc Natl Acad Sci USA. 98:6453–6458. 11344259 10.1073/pnas.101120998 PMC33489

[BHV315C40] Schneider CA, Rasband WS, Eliceiri KW 2012 NIH Image to ImageJ: 25 years of image analysis. Nat Meth. 9:671–675. 10.1038/nmeth.2089 PMC5554542 22930834

[BHV315C41] Seabrook TA, El-Danaf RN, Krahe TE, Fox MA, Guido W 2013 Retinal input regulates the timing of corticogeniculate innervation. J Neurosci. 33:10085–10097. 23761904 10.1523/JNEUROSCI.5271-12.2013 PMC3682386

[BHV315C42] Shatz CJ, Rakic P 1981 The genesis of efferent connections from the visual cortex of the fetal rhesus monkey. J Comp Neurol. 196:287–307. 7217358 10.1002/cne.901960208

[BHV315C43] Sherman SM, Guillery RW 1996 Functional organization of thalamocortical relays. J Neurophysiol. 76:1367–1395. 8890259 10.1152/jn.1996.76.3.1367

[BHV315C44] Sherman SM, Guillery RW 1998 On the actions that one nerve cell can have on another: distinguishing “drivers” from “modulators”. Proc Natl Acad Sci. 95:7121–7126. 9618549 10.1073/pnas.95.12.7121 PMC22761

[BHV315C45] Sherman SM, Guillery RW 2002 The role of the thalamus in the flow of information to the cortex. Philos Trans R Soc Lond B Biol Sci. 357:1695–1708. 12626004 10.1098/rstb.2002.1161 PMC1693087

[BHV315C46] Stafford BK, Sher A, Litke AM, Feldheim DA 2009 Spatial-temporal patterns of retinal waves underlying activity-dependent refinement of retinofugal projections. Neuron. 64:200–212. 19874788 10.1016/j.neuron.2009.09.021 PMC2771121

[BHV315C47] Sun C, Speer CM, Wang GY, Chapman B, Chalupa LM 2008 Epibatidine application in vitro blocks retinal waves without silencing all retinal ganglion cell action potentials in developing retina of the mouse and ferret. J Neurophysiol. 100:3253–3263. 18922954 10.1152/jn.90303.2008 PMC2604840

[BHV315C48] Sur M, Leamey CA 2001 Development and plasticity of cortical areas and networks. Nat Rev Neurosci. 2:251–262. 11283748 10.1038/35067562

[BHV315C49] Sur M, Rubenstein JL 2005 Patterning and plasticity of the cerebral cortex. Science. 310:805–810. 16272112 10.1126/science.1112070

[BHV315C50] Varoqui H, Schäfer MKH, Zhu H, Weihe E, Erickson JD 2002 Identification of the differentiation-associated Na^+^/Pi transporter as a novel vesicular glutamate transporter expressed in a distinct set of glutamatergic synapses. J Neurosci 22 (1):142–155. 11756497 10.1523/JNEUROSCI.22-01-00142.2002 PMC6757588

[BHV315C51] Yamamoto N, López-Bendito G 2012 Shaping brain connections through spontaneous neural activity. Eur J Neurosci. 35:1595–1604. 22607005 10.1111/j.1460-9568.2012.08101.x

[BHV315C52] Ziburkus J, Guido W 2006 Loss of binocular responses and reduced retinal convergence during the period of retinogeniculate axon segregation. J Neurophysiol. 96:2775–2784. 16899631 10.1152/jn.01321.2004

