# Supplementary material for: The Regulation of Corticofugal Fiber Targeting by Retinal Inputs
Source: Cereb Cortex. 2016 Jan 6;26(3):1336–48. doi: 10.1093/cercor/bhv315 (PMC4737616; doi:10.1093/cercor/bhv315)
Supplement: Supplementary Data [file supp_26_3_1336__index.html]

Supplementary Data 

# The Regulation of Corticofugal Fiber Targeting by Retinal Inputs

## Supplementary Data

Supplementary Data

- Supplementary Tables - docx file
- Supplementary Figures - docx file
