# Supplementary material for: The Regulation of Corticofugal Fiber Targeting by Retinal Inputs
Source: Cereb Cortex. 2016 Jan 6;26(3):1336–48. doi: 10.1093/cercor/bhv315 (PMC4737616; doi:10.1093/cercor/bhv315)
Supplement: Supplementary Data [file supp_bhv315_bhv315supp_tables.docx]

|  |  | **Area of control dLGN in mm^2^** ± **s.d.** | **Area of enucleated dLGN in mm^2^** ± **s.d.** | **p** | **n** |
| --- | --- | --- | --- | --- | --- |
| **Golli-τ-eGFP** | **P2** | 1.095 ± 0.03204 | 0.7342 ± 0.06000 | 0.0041 | 3 |
|  | **P4** | 1.179 ± 0.09664 | 0.9316 ± 0.07085 | 0.0003 | 6 |
|  | **P6** | 1.015 ± 0.05616 | 0.7400 ± 0.03643 | 0.0013 | 6 |
|  | **P8** | 1.310 ± 0.1550 | 0.9052 ± 0.06824 | 0.0014 | 4 |
|  | **P10** | 2.196 ± 0.09352 | 1.215 ± 0.1469 | 0.0002 | 5 |
| **Ntsr1-Cre** | **P4** | 1.433 ± 0.08711 | 1.118 ± 0.09820 | 0.0124 | 3 |
|  | **P6** | 1.928 ± 0.2165 | 1.507 ± 0.1124 | 0.0040 | 4 |
|  | **P8** | 2.54 ± 0.4505 | 1.763 ± 0.1473 | 0.0168 | 4 |
| **Rbp4-Cre** | **P4** | 1.836 ± 0.3461 | 1.434 ± 0.1965 | 0.0224 | 3 |
|  | **P6** | 2.122 ± 0.1406 | 1.402 ± 0.09496 | 0.0002 | 4 |
|  | **P8** | 2.114 ± 0.3322 | 1.870 ± 0.08951 | 0.1545 | 3 |

**Supplementary table 1.** Table showing the mean area of control and enucleated dLGN of the Golli-τ-eGFP mice, Ntsr1-Cre::tdTomato mice and Rbp4-Cre::tdTomato mice on coronal sections in mm^2^ ± s.d. A one tailed, paired t-test comparing control dLGN with enucleated dLGN at each age shows that the area of the dLGN was significantly different between control dLGN and enucleated dLGN at all ages, p value shown.

|  | **Area of control dLGN in mm^2^** ± **s.d.** | **Area of dLGN receiving input from injected eye in mm^2^** ± **s.d.** | **p** | **n** |
| --- | --- | --- | --- | --- |
| **P6 epibatidine injection** | 0.9746 ± 0.1297 | 0.8817 ± 0.1052 | 0.0218 | 7 |
| **P6 sterile saline injection** | 1.445 ± 0.2322 | 1.325 ± 0.3119 | 0.1052 | 8 |

**Supplementary table 2.** Area of the dLGN in Golli-τ-eGFP mice on coronal sections in mm^2^ in control dLGN and dLGN receiving input from the eye receiving epibatidine injections or sterile saline injections.
