# Supplementary material for: The Regulation of Corticofugal Fiber Targeting by Retinal Inputs
Source: Cereb Cortex. 2016 Jan 6;26(3):1336–48. doi: 10.1093/cercor/bhv315 (PMC4737616; doi:10.1093/cercor/bhv315)
Supplement: Supplementary Data [file supp_bhv315_bhv315supp_figs.docx]

**
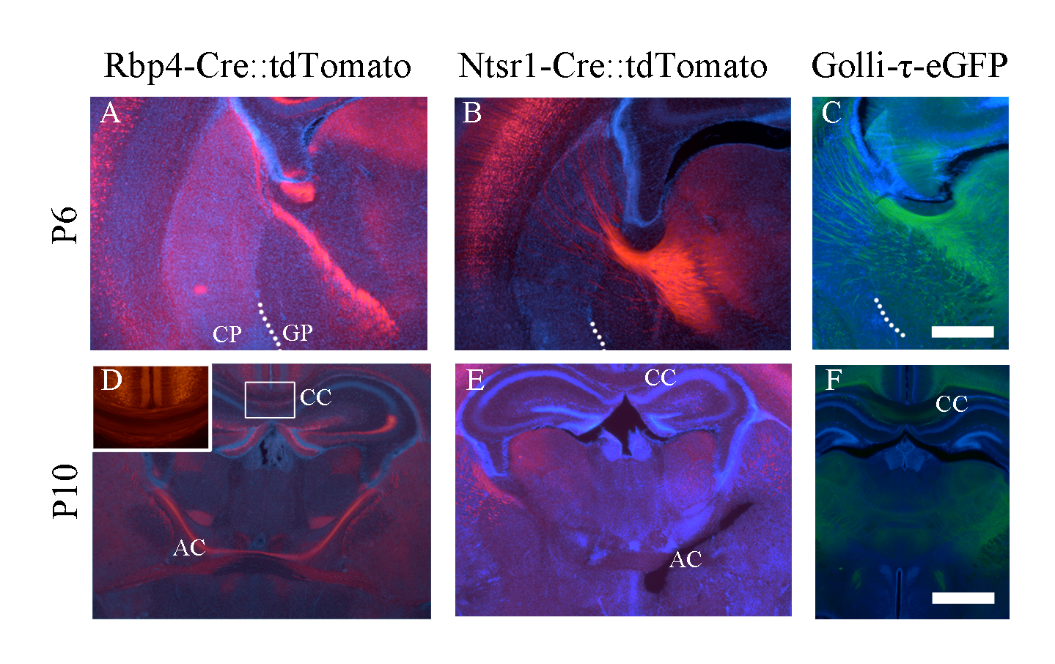
**

**Supplementary Figure 1. Layer V, VIa and VIb have complementary projections to subcortical and inter-hemispheric targets. A.** Layer V Rbp4-Cre::tdTomato fibres project through the caudate putamen of the striatum and narrow to project through the corridor before widening at the cerebral peduncle. Many fibres continue in the cerebral peduncle. Some fibres turn dorsally to enter the thalamus. There is also dense labelling of the caudate putamen. **B.** Ntsr1-Cre::tdTomato layer VI and **C.** Golli-τ-eGFP VI and VIb neurons project through the caudate putamen and narrow to project through the globus pallidus before reorienting to project dorsally into the TRN and thalamus. Dotted line represents boundary between caudate putamen and globus pallidus. **D.** Rbp4-Cre::tdTomato labels cortico-cortical layer V projection neurons whose fibres cross the corpus callosum (CC) and the anterior commissure (AC). **E.** Ntsr1-Cre::tdTomato does not label fibres in the corpus callosum or anterior commissure. **F.** Golli-τ-eGFP labels cross-callosal intracortical fibres. Abbreviations used: CP, caudate putamen; GP, globus pallidus; AC, anterior commissure; CC, corpus callosum. Scale bars= 500µm (A-C), 250µm (D-F). n= 3 per strain per age.


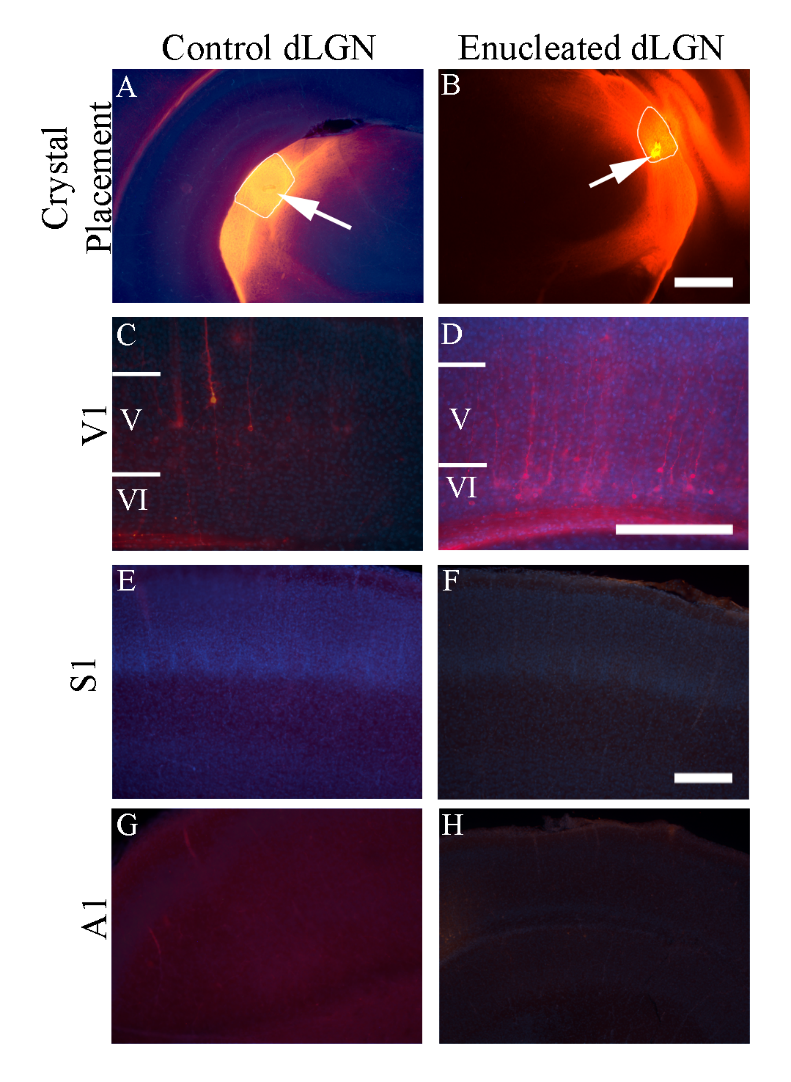


**Supplementary Figure 2. Fibres prematurely entering the dLGN are from primary visual cortex shown by retrograde labelling from the dLGN. A-B.** Representative DiI crystal placements in the control and enucleated dLGN. **C.** Sparse back-labelled cells were sometimes visible in layer V of the control primary visual cortex n=4. **D.** Back-labelled cells were present in layer VI of the enucleated primary visual cortex n=3. No cells were found back-labelled in the control or enucleated primary somatosensory cortex (**E and F**) or the control or enucleated primary auditory cortex (**G and H**). Scale bars= 500µm A-B, 250µm C-D, E-H.

**
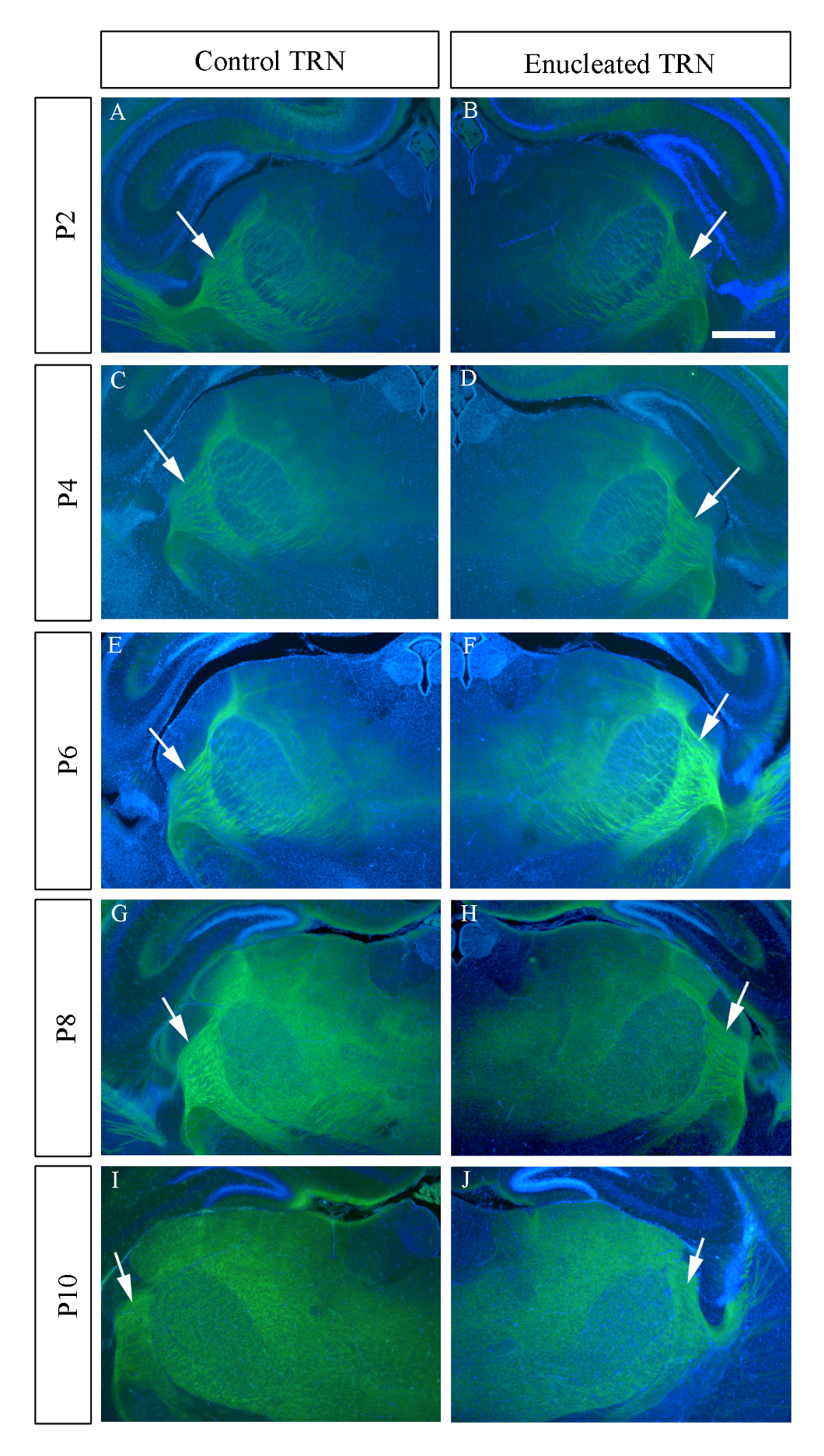
**

**Supplementary Figure 3. Ingrowth of Golli-τ-eGFP layer VIa and VIb fibres to the visual sector of the TRN in the control hemisphere and enucleated hemisphere. A-J.** Golli-τ-eGFP fibres project to the visual sector of the TRN (white arrows). The patterning and timing of Golli-τ-eGFP fibre ingrowth to the TRN is not different between the control and enucleated hemisphere. P2 n=3, P4 n=6, P6 n=6, P8 n= 4, P10 n=5. Scale bar= 500µm.


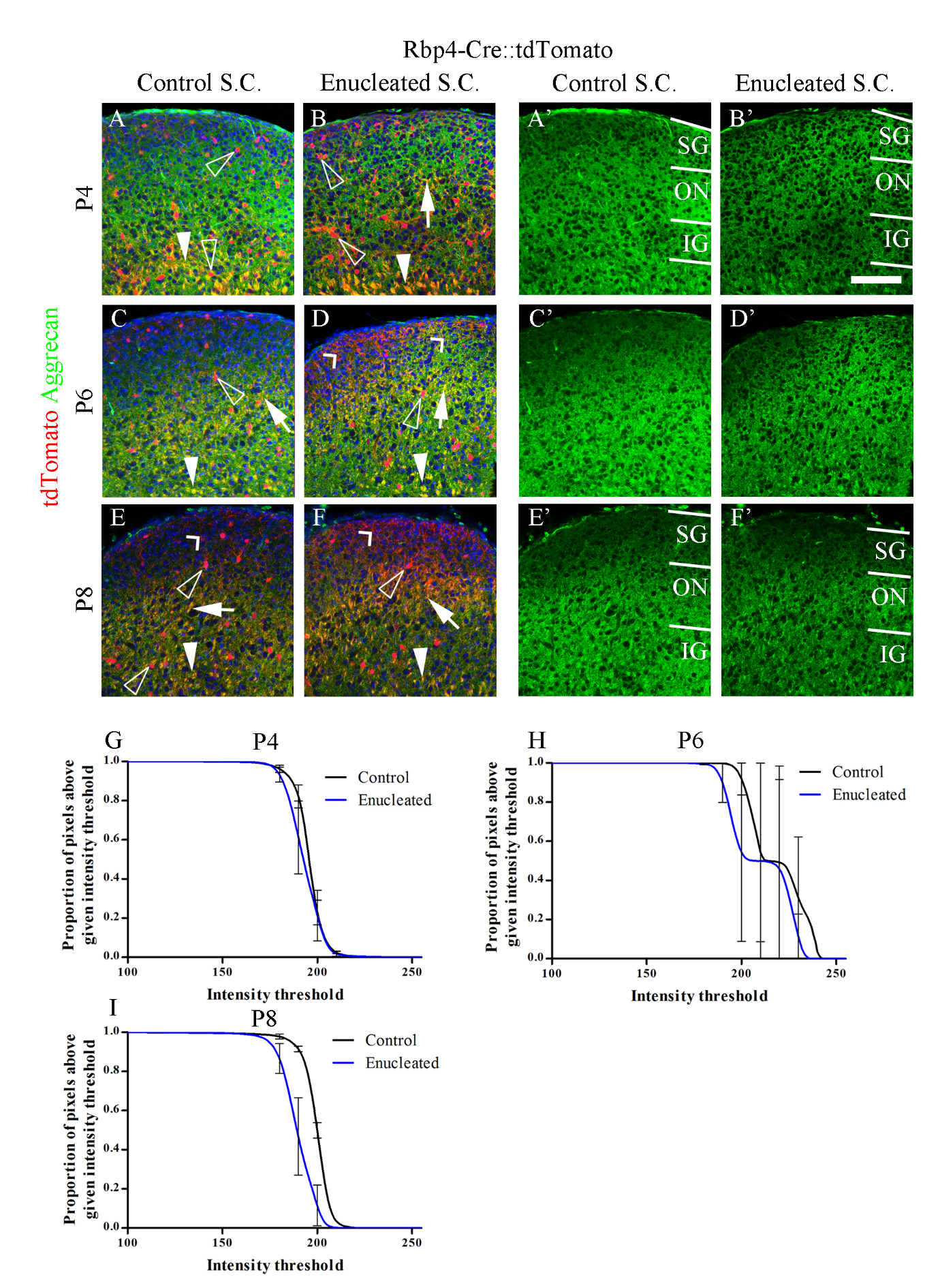


**Supplementary Figure 4. Aggrecan immunohistochemistry in the superior colliculus following monocular enucleation at birth. A-F.** Aggrecan is present in the control and enucleated superior colliculus. It co-localises with Rbp4-Cre::tdTomato positive layer V bundles in the intermediate white layer (filled arrow heads) and fibres in the optic nerve layer (filled arrow). It does not co-localise with tdTomato expressing cells which reside in the superior colliculus (hollow arrow heads). At P6 and P8 it co-localises with tdTomato positive fibres in the superficial grey layer (chevron arrows **D-F**). **A’ and B’.** At P4 aggrecan labelling is not different between the control and enucleated superior colliculus n=3. **C’ and D’.** By P6 aggrecan labelling is weaker in the superficial grey layer of the control and enucleated superior colliculus although there is a stronger patch in the lateral side of the enucleated superficial grey layer n=4. **E’ and F’.** By P8 aggrecan labelling is absent in the control and enucleated superficial grey layer but present throughout the rest of the superior colliculus n=3. Midline is on the right of control images and on the left of enucleated images. **G, H and I.** Pixel intensity analysis of the superficial grey layer shows no significant difference between the intensity of aggrecan staining in the control and enucleated hemispheres at P4 (**G**) P6 (**H**) and P8 (**I**)**.** Scale bar= 50µm.
